# What makes advocacy work? Stakeholders’ voices and insights from prioritisation of maternal and child health programme in Nigeria

**DOI:** 10.1186/s12913-020-05734-0

**Published:** 2020-09-18

**Authors:** Benjamin Uzochukwu, Chioma Onyedinma, Chinyere Okeke, Obinna Onwujekwe, Ana Manzano, Bassey Ebenso, Enyi Etiaba, Nkoli Ezuma, Tolib Mirzoev

**Affiliations:** 1 Department of Community Medicine, College of Medicine, University of Nigeria Enugu Campus, Enugu, Nigeria; 2 Department of Community Medicine, University of Nigeria Teaching Hospital Enugu, Enugu, Nigeria; 3 Department of Health Administration and Management College of Medicine, University of Nigeria Enugu Campus, Enugu, Nigeria; 4 School of Sociology & Social Policy, University of Leeds, Leeds, UK; 5 Nuffield Centre for International Health and Development, University of Leeds, Worsley Building, Clarendon Way, Leeds, UK; 6 Health Policy Research Group (HPRG), College of Medicine, University of Nigeria, Enugu Campus, Enugu, Nigeria

**Keywords:** Advocacy, Realist evaluation, Maternal and child health, Nigeria

## Abstract

**Background:**

The Nigerian government introduced and implemented a health programme to improve maternal and child health (MCH) called Subsidy Reinvestment and Empowerment programme for MCH (SURE-P/MCH). It ran from 2012 and ended abruptly in 2015 and was followed by increased advocacy for sustaining the MCH (antenatal, delivery, postnatal and immunization) services as a policy priority. Advocacy is important in allowing social voice, facilitating prioritization, and bringing different forces/actors together. Therefore, the study set out to understand how advocacy works - through understanding what effective advocacy implementation processes comprise and what mechanisms are triggered by which contexts to produce the intended outcomes.

**Methods:**

The study used a Realist Evaluation design through a mixed quantitative and qualitative methods case study approach. The programme theory (PT) was developed from three substantive social theories (power politics, media influence communication theory, and the three-streams theory of agenda-setting), data and programme design documentation, and subsequently tested. We report information from 22 key informant interviews including national and State policy and law makers, policy implementers, CSOs, Development partners, NGOs, health professional groups, and media practitioners and review of relevant documents on advocacy events post-SURE-P.

**Results:**

Key advocacy organizations and individuals including health professional groups, the media, civil society organizations, powerful individuals, and policymakers were involved in advocacy activities. The nature of their engagement included organizing workshops, symposiums, town hall meetings, individual meetings, press conferences, demonstrations, and engagements with media. Effective advocacy mechanism involved alliance brokering to increase influence, the media supporting and engaging in advocacy, and the use of champions, influencers, and spouses (Leadership and Elite Gendered Power Dynamics). The key contextual influences which determined the effectiveness of advocacy measures for MCH included the political cycle, availability of evidence on the issue, networking with powerful and interested champions, and alliance building in advocacy. All these enhanced the entrenchment of MCH on the political and financial agenda at the State and Federal levels.

**Conclusions:**

Our result suggest that advocacy can be a useful tool to bring together different forces by allowing expression of voices and ensuring accountability of different actors including policymakers. In the context of poor health outcomes, interest from policymakers and politicians in MCH, combined with advocacy from key policy actors armed with evidence, can improve prioritization and sustained implementation of MCH services.

## Background

The World Health Organization describes advocacy for health as a combination of individual and social actions that are expected to achieve social acceptance, political commitment, policy, and systems support for a given health goal or programme [1]. This can include many activities that a person or organization undertakes including media campaigns, public speaking, commissioning and publishing research, capacity building, relationship building, forming networks, and leadership development. The main goals that underpin health advocacy include empowerment of the disadvantaged (facilitational advocacy) and systems support and protection of the vulnerable (representational advocacy) for a particular health goal or programme [2]. Governments create policies, which successive governments and international agencies must adhere to while implementing these policies. Most health-promoting organizations either advocate for new policies or the implementation of already formulated policies, especially when they are not complying with the laid down guidelines [3]. Thus, advocacy in the domain of maternal and child health (MCH) which is health service provided to all women in their reproductive age groups, i.e., 15–49 years of age, children, school-age population and adolescents [4], is necessary for ensuring that political leaders consider MCH issues important enough to attract the provision of resources appropriate with the severity of the problem and ultimately improving the provision of MCH services to contribute to improved health outcomes. These MCH issues include a very low skilled birth attendant, limited access to MCH services due to poverty, non- functional facilities, poor access roads, ignorance, cultural taboos, etc., leading to high maternal and child mortality [5].

In Nigeria, maternal and child health indices have remained poor and the observed outcomes have been partly attributed to the persistent low coverage and uptake of MCH interventions [3]. For example, in 2018, only 67% of pregnant women were able to receive antenatal care (ANC) while 43% of mothers delivered with a skilled birth attendant [6]. Strategies adopted by the Federal Government to improve MCH indices have thus focused on broadening access to MCH services and improving health outcomes among these population groups. For example, in 2009, the Federal Government established the Midwives Service Scheme (MSS) to address the barriers created by the inequitable access to skilled care, especially among disadvantaged population groups [7].

In 2012, a social protection programme called Subsidy Reinvestment and Empowerment Programme (SURE –P) was established by the federal government to mitigate the immediate impact of the partial removal of petroleum subsidy on the population. The intervention areas of the SURE-P are categorized into two, social safety net projects and infrastructure development projects. The Maternal and Child Health (MCH) Programme is the first programme under the Social Safety Net Projects in the (SURE-P/MCH) established under the authority of the Federal Ministry of Health and implemented by the National Primary Health Care Development Agency (NPHCDA) to improve health indices of maternal and child care especially in rural communities [8]. SURE-P/MCH comprised supply and demand components. The supply component included recruitment and training of staff (2000 midwives, 10,000 community health workers), infrastructure development, improving availability of supplies and medicines, and activation of Ward Development Committees. The demand component aimed to increase utilisation of MCH services during pregnancy and at birth using a conditional cash transfer (CCT) programme as a resource [8].

However, in 2015 the newly elected national government suspended funding to the programme after 47 months of its implementation. Following the end of the SURE-P/MCH, there have been increased efforts from various key stakeholders to ensure and sustain the prioritization of MCH (antenatal, delivery, postnatal and immunization) services as a policy priority through different advocacy and lobbying activities. Thus the federal government through the federal ministry of health (FMOH) emphasized that MCH was a key focus area of the ministry’s agenda to revitalize primary health care (PHC) [9–11]. Therefore, despite the suspension of funding to SURE-P, the federal and some state governments continued to implement other free MCH interventions at PHC centres. The free MCH programme (FMCHP) was implemented in 12 states in Nigeria by the National Health Insurance Scheme (NHIS), between 2009 and 2015, using funds from the debt relief gains [12]. These states included Bayelsa, Bauchi, Cross River, Gombe, Imo, Jigawa, Katsina, Niger, Ondo, Oyo, Sokoto, and Yobe. There was also the MSS implemented by the National Primary Health Care Development Agency (NPHCDA) [7], and the Saving newborn lives programme of the federal ministry of health [13]. Some states also implemented their free MCH programmes for example in Enugu state [14] and Jigawa state [15].

The third sector (i.e organisations that are neither public nor private sector including voluntary and community organisations, social enterprises, mutuals and co-operatives, the first 2 sectors being public and private organizations), operating in sub-Saharan Africa often focus on political advocacy to change policy negotiations and implementation [16] using a combination of top-down and bottom-up activities [17]. Also, civil society organizations CSOs-organised civil society which can be informal and formal entities such as non-governmental organisations (NGOs), CBOs, faith-based organisations (FBOs) require a lot of advocacies to the public sector and the politicians to achieve the change they need for the masses. It is important to note that these organizations were the purveyors of advocacy and not the providers of services.

There were ongoing advocacy initiatives by other bodies, including WHO Partnership for maternal, neonatal, and child health (PMNCH) conference [18, 19] after the cessation of the SURE-P/ MCH programme. For example, the PMNCH were advocating for increased allocation of MNCH resources at Federal, State and Local levels [20], supported health investments including those made through the Global Financing Facility for the Global Strategy for Women’s and Children’s Health (2010–2015 and 2016–2030) and the “Every Woman Every Child” movement through the Global Financing Facility (GFF) [21]. Despite, these advocacy initiatives, the role and the effectiveness of the advocacy by different groups in ensuring the sustenance of the MCH programme were unexplored with scarce local research in this area.

In the past, several capable individuals with rich personal networks in government and civil society organizations had promoted the safe motherhood cause in Nigeria by engaging in advocacy activities but the effectiveness of such measures is uncertain [22]. It has been stated that priority for MCH programme is present in political agendas in low and middle-income countries (LMIC) when: the government, enacts policies that address the problem; political leaders are interested in the issue; and the government allocates and releases funds to tackle the problem [22] Also, according to the Kingdon’s three-streams theory of agenda-setting, for an issue to be placed on the policy agenda, the three streams need to converge at the right moment [23]. Advocacy helps contribute to the problem stream by emphasizing the severity of the issue, and the politics and policy streams by attracting the attention of politicians and policymakers and linking the issues to relevant international and national frameworks – thus ultimately contributing towards convergence of three streams of agenda-setting. But the role of advocacy in ensuring the achievement of all these is not well documented.

### Theoretical framework

Issues around Advocacy formed one of the eight programme theories (PT) which were explored in the wider study mentioned shortly in the methods section. The development and testing of the PT drew upon three substantive social science theories that help understand advocacy: the theory of power politics [24], media influence communication theory [25], and the three-streams theory of agenda-setting [23].

The Power Politics theory, also known as Political Elites or Power Elites theory, proposes that the power to influence policy is concentrated in the hands of a few [26]. Some people have more power than others. Policy change is therefore made by working directly with those with the power to make decisions or influence decision making. This theory is useful when there is one or more key allies in a position of power on the issue and focus may be on incremental administrative or rule changes. The strategies to influence change include direct advocacy to key decision-makers and/or influentials when policy opportunities emerge and developing relationships with decision-makers and/or influentials.

The Max McCombs and Donald Shaw’s Media Influence theory, suggests that Political issues being on the public’s agenda will depend on the extent of coverage a given issue receives by mass news media [26]. Political issues that are salient and ever-present in the media tend to be the same issues that the public have awareness of and consider key. Some of the underlying assumptions are that the news media is generally one’s primary source of political information. This theory is useful when there is a strong media-related capacity and need to put the issue on the radar of the broader public. The strategies to influence change are that the media conduct media advocacy campaigns (e.g., write letters to the editors, editorials, or press releases; hold public events; disseminate research).

The Kingdon’s three-streams theory of agenda-setting notes that policy can be changed during a window of opportunity when advocates can successfully connect two or more components of the policy process (e.g., the way a problem is defined, the policy solution to the problem, and/or the political climate of their issue). This is useful when one can address multiple streams simultaneously (e.g., problem definition, policy solutions, and/or political climate) and there is the internal capacity to create, identify, and act on policy windows [26]. The strategies here include defining the problem, developing policy solutions, strengthening organizational capacity, and influencing the political climate, e.g. coalition building and demonstrations.

This study, therefore, set out to understand how advocacy works - through understanding what comprise effective advocacy implementation processes and what mechanisms (i.e. reasoning and resources) are triggered by which contexts to produce the intended outcome (increased political prioritization of the MCH). Even though this is intended as a research paper, this manuscript also carries an implicit advocacy objective. We hope documenting how concerned individuals and groups, armed with evidence advocate to the government through policy influencers/champions, national and local media, will help key decision-makers to understand the severity of the problem and will encourage government commitment and lead to increased enactment and funding of sustainable MCH policies.

## Methods

This paper is a component of a study titled “Determinants of effectiveness and sustainability of a novel community health workers programme in improving MCH in Nigeria”. In this study. The sudden withdrawal of SURE-P is used as an explanatory case study [27] to explore cause-effect relationships of advocacy activities in MCH within the Nigerian context using Anambra State as a case. Anambra state was identified in consultation with the Federal and State Ministry of Health (MOH) and the SURE-P national team lead [28]. Thus Anambra state was used for the advocacy study as advocacy activities concentrated mostly at the national level and needed to show that events were also taking place at the subnational level but none at the local governments.

The study used realist evaluation through mixed-methods approach, as described in another study [28]. Realist Evaluation is based on the supposition that interventions constitute ideas and assumptions (programme theories), about how and why they are expected to work [29]. It is a theory-driven approach that involves developing, testing, and refining specific programme theories (PTs).

The authors first conducted a literature and document review of MNCH advocacy activities carried out after the SURE-P programme ended. This review included a systematic search and synthesis of published peer-reviewed articles, reports and articles from agencies and research studies, and news stories. The objective of the advocacy process was to sensitize stakeholders on the need to keep MCH services (antenatal, delivery, postnatal and immunization) on the political and financial agenda and our purpose was to map changes in policy and programme environments at federal and state levels as well as mapping advocacy and lobbying events that helped to keep MCH on the political agenda. The search and data extraction were done by two of the authors using a proforma (see Table 1). The headings of the proforma included advocacy event and why; person/group who led event; date and venue of event; contextual features of the event; mechanism (What made the event work); the outcome of the event (e.g. what was the effect of advocacy and lobbying). Advocacy issues formed one of the eight PTs which were initially developed from the literature, document review and consultations with key policy actors, and then were empirically tested, validated, and refined. This led to the identification of the advocacy issues used to develop the initial programme theory (gleaned from the mapping of advocacy/policy timelines and relevant literature).

**Table 1:** Mapped advocacy events at the national and Anambra State (2015–2017)

S/N	Event and Purpose	Who led the event	Date and venue	Key context and mechanisms	Outcome
1.	March by market women and communities in northern Nigeria against the stoppage of SURE-P	Community leaders	11/2015 at (FMoH) Abuja	Police stopped the protesters several times, but the influential community leaders who the police could not arrest/detain led the march	The Minister set up a committee to revitalise the MSS programme that contained activities similar to SURE-P
2.	The 9th International Congress, 49th Annual General Meeting And 50th Anniversary Celebration Of Society Of Gynaecology And Obstetrics Of Nigeria (SOGON)	SOGON	24–27/11/ 2015. Ladi Kwali Conference Centre, Sheraton Abuja	Theme: Promoting Women’s Health in Nigeria. Multi-stakeholder participation by Health Professionals, Politicians, Policymakers including the Minister, Lawyers, Civil Society, Development partners, Pharmaceutical and Allied companies and Mass Media	Awards to Policy elites and maternal health advocates
3.	Inaugural summit on accountability for reproductive, maternal, newborn, child and adolescent health (RMNCAH)	FMoH in collaboration with Champions for Change, the Health Reform Foundation (HERFON) of Nigeria, and Women Friendly Initiative	16–18/2/2016 Abuja, Nigeria.	Engagement of multiple stakeholders in health to discuss the status of RMNCAH in Nigeria and need to strengthen it	The signing of a declaration by participating organizations calling for action by the Government to address maternal and neonatal deaths and declaring them a priority. A task force was set up by FMoH to ensure maternal mortality reduction with a target to increase funding for family planning services from US $3million to US $4million, from 2018.
4.	Advocacy to media to report issues surrounding Health budget and finance in media space	HERFON	3/ 2017, Lagos	Symposium for Health writers’ Association of Nigeria (HEWAN). Media’s power in ensuring accountability and good governance and interaction between an advocacy group and the media	HEWAN sensitized and mobilized on accountability for health funds. More reports in the media on accountability for health
5.	Press conference by a group of NGOs on the shortfall in the budgetary allocation to the health sector	the National Association of Community Health Practitioners, Development Research and Project Center and the Partnership for Advocacy in Child and Family at Scale	11/2017 at Abuja Nigeria.	A collaboration of 3 NGOs stressing for media support to holding government accountable for adequate funding of the health sector and full implementation of key policies that will enhance child and family health in Nigeria.	Awareness creation on the budget short-fall precipitating the appropriation of the Basic Health Care Provision Fund (BHCPF) in the 2018 national budget
6.	A symposium on the role of media in advocating for increased health sector budget in Nigeria.	Health Writer’s Association of Nigeria (HEWAN)	4/2017 Lagos State.	Organized by the media and an address delivered by Health Reform Foundation (HERFON) on the need to appropriate the BHCPF in the budget to improve funding of PHC and MCH	The media promised more commitment to reporting MCH issues. This precipitated the appropriation of the BHCPF in the 2018 national budget
7.	Meeting of governors’ wives for the support of the Reproductive, Maternal, Newborn Child, Adolescent Health + Nutrition (RMNCAH+N) services	The wife of the Nigerian president	11/2016 in Abuja,	The wife of the president, as well as the minister for health, addressed the governors’ wife forum on the need to support (RMNCAH+N) services in their various states	Developmental partners, the private sector, and the government enjoined to provide support to the activities governors’ wives coalition. This made the state governors prioritize RMNCAH+N
8.	4th National family planning conference	Nigerian minister for health	7–9/11/2016 Abuja	Theme: Family Planning in Nigeria: The Journey so far, hosted by the United Nations. Health Minister encouraged Nigerians to engage in family planning stressing that neither Christianity nor Islam disallowed it.	The masses got enlightened on family planning. The FMoH and development partners got committed to Family planning issues with a promise to release more funds.
9.	Workshop on Saving One Million Lives Program For Results (SOML PforR) and CSO Roundtable conference	Connected Development - CODE (an NGO)	13/4/ 2017 Abuja	Recognition of the need for CSOs to be carried along in the implementation processes of (SOML PforR) and the need for accountability. Attendance by FMoH, the World Bank, and CSOs enhanced the workshop	Engender trust and the values of Open Government in the Nigerian society. Reinforced the need for accountability of the funds released to states in 2016 and results
10.	Aisha Buhari urges Governors’ Wives to champion reproductive issues	The wife of the Nigerian President	10/10/ 2017. Abuja.	A need to reduce maternal mortality, child malnutrition and child mortality in under-five children. Participation of the Federal Ministry of Health, UNICEF, wives of the 36 state Governors and National Primary Health Care Development Agency, Development partners	The Minister for Health pledged the Government’s support for the program on reproductive health. The Governor’s wives committed to partner with the wife of the President in implementing programmes to reduce maternal and child mortality in their respective states. This precipitated the appropriation of the BHCPF in the 2018 National Budget and release of the Saving One Million Lives funds to States
11.	Public, private sector and CSO engagement to achieve SDG targets for reproductive, maternal, child health	Private Sector Health Alliance of Nigeria, (PHN) and Nigerian Integrated Coalition for Improving RMNCAH (NICIR)	19/12/2017 Abuja	Identification of opportunities for synergies/collaboration between public and private health sector players. Coming together of many organizations including the United Nations’ Every Woman Every Child initiative; Merck for Mothers; Nigeria Global Financing Facility (GFF) and presentation of diverse, but unique perspectives for improving RMNCAH service delivery	This facilitated the release of GFF funds for RMNCAH
12.	The National Summit on RMNCAH to address accountability in healthcare delivery for women, newborns, and other vulnerable groups	Champions for Change, HERFON and Women Friendly Initiative	16–18/2/ 2016 Abuja	Discussion on the new 2030 SDGs, and examination of how effective ongoing efforts are in delivering interventions to women and children in Nigeria. Collaboration between many strong CSOs, FMoH, WHO, and UNICEF enhanced the summit.	Alliances across the public and private sector media, and religious institutions for the prioritization of MCH
13.	Tour of primary health centres by the wife of the Anambra State Governor	Wife of the Anambra State Governor	11/2016. Anambra State, Nigeria	Promotion of health and safe delivery practices for pregnant women and the tour had the support of the State government and Chairmen of the Local Government Areas.	Distribution of maternal delivery kits (MAMA KIT) to pregnant women present. Facilitation of the passage of the State Primary Health Care Development Agency bill and the release of counterpart funds for the Basic Health Care provision Fund in Anambra state in 2018 for the provision of basic minimum health package.
14.	Mothers’ Summit on getting priorities right for a peaceful home and society.	An NGO, “Caring Family Enhancement Initiative, CAFÉ, for self-reliant”.	10/9/2017 Prof. Dora Akunyili Women Development Centre, Awka Anambra State.	The need to be meaningfully engaged and earn an income to support the upkeep of the family including health bills and child nutrition. Support by the wife of the Governor, Governor of Anambra state and presence of mothers from the 179 communities of the state ensured the success of the event	Presentation of empowerment equipment like Garri and Palm Oil processing machines to women cooperative societies by the Governor

The advocacy issues that guided this PT were *“In the context of poor health outcomes, interest from policymakers and politicians in maternal and child health care (MCH), combined with advocacy and lobbying from key policy actors to prioritise MCH, is likely to help generate and maintain political and economic commitment ultimately contributing to sustained implementation of and access to MCH services for vulnerable groups”* A total of 14 advocacy events at the National and Anambra State levels related to changes in policy and programme environment were mapped during theory testing.

Next, we sought to develop an in-depth understanding of the experiences and practices of advocacy groups at the national and state level and this provided a range and depth of experiences that were relevant to our phenomena of interest. Using purposive sampling methods, we developed the list of respondents for interviews based on their roles in advocacy events. These roles included organizational leads and key individuals spearheading the advocacy combined with policymakers who were on the ‘receiving end’ of advocacy.

The document review and tracking of advocacy events in MCH in Nigeria informed our selection of the respondents at the Federal level and in Anambra State (the study state for the larger project to understand what happened at the sub-national level). They included 22 in-depth interviews (IDIs) with stakeholders (a stakeholder being a person, group or organization that has interest or concern in the issue at hand and in this advocacy case, they are the government, the policymakers, the public servants (eg. FMOH), the CSOs, the international organizations, the media, the professional groups and representatives of the community). On the whole, 3 CSOs, 3 Development Partners, 3 NGOs, 2 health professional groups, 3 media practitioners, and 8 policy-makers (5 from the National and 3 from the State level) all of who were active in advocacy events were selected. They were also selected to reflect differences in groups, occupations, and professional backgrounds. Using an IDI guide, they were interviewed by 4 interviewers. This gave the details of the activities they carried out, the output and outcome as they continued advocating for these until the desired effect was achieved.

The IDIs were semi-structured around our programme theory to validate, test, and refine it using a topic guide (see Additional file) [30]. We developed the semi-structured interview guide around the programme theory because we needed to conform with the realist evaluation methodology where the initial ‘program theories are formed from the findings of the literature review, then a guide is developed to ask questions that will either confirm or disprove the findings of the first theory i.e. the gleaning stage.

This included the context of MCH in Nigeria and how actors perceived maternal health as a problem, the strategies adopted by the actors, the outcome of the advocacy, and what enabled or constrained the advocacy events. The interview guides were different for the producers and users of advocacy and designed to focus on each group’s strength, though they were also asked to corroborate that they knew what the other group was doing.

All interviews were undertaken in person in English generally after written informed consent was obtained from all respondents. All interviews were also conducted in the participants’ offices, were audio-recorded and transcribed verbatim by professional transcribers for analysis. To ensure quality, we used the realist and meta-narrative evidence synthesis (RAMESES) publication standards [31] for reporting realist synthesis as quality assurance checks within our study. This recommends in line with a realist approach, that existing theory is mixed with the developed PT to enhance the explanatory endeavour of the study. Also, the quality was ensured at different steps of the process (piloting and post-piloting revision of tools, collection, transcription, translation, anonymization, digitization/entry into software, coding, and analysis). Mechanisms for quality assurance used included appropriate training (e.g. of transcribers of key concepts/terms used), multiple researchers working on the same data (e.g. coding by at least two researchers), continuous peer-review and peer-support within and between the different partner teams.

### Data analysis

Retroductive approach to analysis [32] was used which involved continuous engagements and refining of the theory against the data and the existing literature on the subject. Qualitative data recordings were transcribed verbatim, anonymised, double coded in MS Word using colour-coded highlights and, analysed using manual thematic and framework analysis of the main topics outlined in the interview guide. Other codes not included in the guide emerged during the reading of the interviews. Findings were supplemented and validated with document review. The combination of three substantive theories of power politics, media influence communication theory and the three-streams theory of agenda-setting was used to infer causal relationships within certain circumstances.

## Results

### Agenda setting and community sensitization in MCH

The changes in policy and programme environments that help to keep MCH on the political agenda included changes at the federal level, influences in Anambra state, and events in other states of the country that include Anambra state. From the mapping, a total of 14 events were implemented of which 2 were at the sub-national/state level and 12 were at the federal level. As shown in Table 1, key advocacy organisations and individuals included health professional groups, the media, civil society organisations and NGOs with similar objectives coming together informally for MCH issues, powerful individuals, and policymakers. The nature of their engagement included organizing demonstrations, workshops, symposiums, town hall meetings at the national level, individual meetings, press conferences, and engagements with media (see Table 1).

Despite remaining national and international priority, sustaining citizens’ interests, political and financial commitment to MCH services in Nigeria often requires effective advocacy efforts. We found that key outcomes of advocacy included financial commitment, political involvement, policy enactment, and implementation as shown in Table 1. Specifically, the outcomes included the reactivation of the Midwives service scheme (MSS), which was in place before the advent of SURE P/MCH, appropriation of the Basic Health Care Provision Fund (BHCPF) in the 2018 national health budget, signing of a declaration by participating organizations calling for action by the Government to address among other things, maternal and neonatal death and declaring them a priority and leading to the federal government setting up a task-force to speed up the reduction of maternal deaths with a target to increase funding for family planning services from US $3million to US $4million, from 2018, media sensitization on accountability for health funds. Other outcomes included prioritization of Reproductive, Maternal, Newborn Child, Adolescent Health plus Nutrition (RMNCAH+N). Also, the World Bank Approved US$500 Million to improve MCH, achieve the ‘Saving One Million Lives’ Goal (a high-impact reproductive and child health and nutrition interventions) whose operation was expected to last from August 1, 2015, to December 2019 [33]. It is important to note that although the World Bank offers packages, advocacy enabled the government to agree to seek those packages and use the funds correctly.

There was raised awareness and ‘education’ of the State governor about the significance of health issues through advocacy. According to one of the respondents, *“Advocacy is a powerful tool because most of these people, they are not health workers, the governor is not a medical doctor, so it is not like he doesn’t know, but when you come to him as an advocate and you can give him facts, looking at indices and looking at what is on the ground, telling him the gaps and everything, he will understand and he will quickly key into it”. (Policy Maker State).*

Some civil society organizations (CSOs) in Nigeria alluded to having achieved a lot for MCH by advocating government and other relevant stakeholders: *“Our organization appreciates the nature and importance of advocacy and that is one of the cardinal things we do with very good results. Like we advocate the government, and the State governors especially the governors’ wives in some states because many are interested in knowing what is happening in their state” (Professional Group, National).* Different actors were targeted differently in different states, for instance, in some areas “governors’ wives” were targeted since they seemed to act as knowledge brokers to other elite decision-makers.

Multiple factors impact the potential of advocacy to generate change [34] in MCH policies such as, the topic, the political time and the socio-economic context, and the type and coalitions of organisations involved in the campaigns but some respondents felt they could have a direct impact, for example, in the case of Nigeria Every New Born Action Plan (NIENAP). A respondent noted that *“UNICEF was interested in maternal and child nutrition and when the benefit package was developed, it didn’t have anything on nutrition because they wanted a slim benefit package, but there was this targeted advocacy to the Minister of Health and the Minister of Finance and eventually it was agreed to add nutrition to the benefits package” (Policy Maker, National).* The individual who represented UNICEF was able to convince the ministries of health and finance of the importance of MCH and thus conferred international legitimacy, credibility, power, and recognition as mechanisms through which advocacy worked on this occasion. Another example was the passage of the State Primary Health Care Development Agency bill in Anambra state. This led to Anambra State releasing their counterpart fund for the Basic Health Care Provision (BHCPF), and accessing the main fund from the Federal Ministry of Health for the delivery of the Basic Minimum Package of Health Services, including basic emergency obstetric and newborn care (BEmONC) in 2019. The persistence of the CSOs and the timing/message convinced the governor to take this forward. This was captured by a respondent thus:

*“ … … we championed it and paid advocacy visit to the house of assembly and the commissioner for health then and the governor took it upon himself to send the bill as an executive bill to the house of assembly. And after advocating to even the ministry of justice and other line ministries, it was passed. And then we persevered and after some time, the State Agency was inaugurated and members were appointed and inaugurated immediately and they moved into action” (CSO State).*

According to the respondents at the sub-national level, where some groups like the CSOs kept advocating and checking the budgets and releases to the MNCH sector, advocacy has also led to an increase in funding for MCH at the sub-national level, for example, the increased package of health services for mothers and children in the current Basic Healthcare Provision Fund (BHCPF) was due to advocacy and the increased releases in budget funds at the state level was also attributed to advocacy by some groups. The release of their counterpart funding for the BHCPF was also due to better awareness of the value of social sector investments and possibly the ability to demonstrate visible political gains (which will help them get re-elected). Advocacy is an explicit aim in some local NGOs as this participant explained *“advocacy has always been an integral part of our programme management. Over the years the state government has tried to increase the budget from what it used to be up to where we are now as the elections are just by the corner … And so I can say that the increase in the budget was as a result of that advocacy and the subsequent advocacy that happened in the past. So eventually, the 2018 budget for health was increased” (NGO State).*

### Contextual factors and mechanism of advocacy in MCH

The key contextual influences which determined the effectiveness of advocacy measures for MCH include the political cycle (given the change that comes with MCH interventions with a change in government), availability of evidence on the issue, networking with powerful and interested champions, and alliance building in advocacy.

#### Spatiotemporal factors: timing and the political cycle in Nigeria

Change in government can determine the sustainability of an MCH programme. For example, the change in government led to the termination of the SURE-P and a change in the direction of MCH policy as explained by one of our respondents. *“One of the biggest problems in Nigeria has been issues of governance and policy inconsistency, and these inconsistencies are coming by the cycle of democratic governance in Nigeria. So, when you change the government, their priorities automatically change, their attention changes and so their political economy shapes what you are doing, and the politics around what you are doing” (Development partner).*

At the sub-national/ State level, the change of power at the national level also led to a changed direction. This was captured by a respondent thus: *“actually, you know that most times the government policy comes and if there is somebody that is driving it and that person goes out, the person that comes in though he will inherit assets and liability, may not be interested in that programme. He will look for the one that he will initiate” (CSO State level).* On the other hand, change in the political cycle can create opportunities for advocacy., When a new government has a vision in some areas in health, decision-makers are more likely to listen to the advocates because *“they are liable then and can listen to suggestions and are more willing to impress the people” (Media State level).*

It was also noted that to be more effective, advocacy needs to be timely, strategic, and sustained. It is needless starting advocacy when it is known that the tenure of the government is going to end soonest because it is going to be a waste of resources. According to a respondent, advocacy *“has to be well-timed. For instance, if I’m working in a state and I know the governor is completing his tenure in 2 months, I will have to wait for the incoming one … ..it will be a waste of resources if I’m going to advocate … … it means my advocacy is not well-timed if I do so. I will rather wait until the new governor comes in because in any transition you need to be mindful of how you invest in advocacy” (Development partner).*

#### The role of evidence: knowledge production and brokerage in MCH

The availability of credible and convincing evidence is the key to successful advocacy. For example, evidence was identified as significant in the implementation of the free MCH services in Anambra State. Powerful videos of graphic images used for advocacy triggered a sense of sympathy, fear of civil unrest/media coverage) which then contributed to better responses to the MCH issues by the government. As noted by a respondent, *“When we visited the governor, we showed him videos of how people were delivering with some people putting herbs inside somebody’s body parts, by jumping on somebody’s tummy to push out the baby. All these things have been captured by the videos, and how people died, and so on” (Health Professional Group).* Thus the policy champions relied on their reputation of having extensive experience in maternal health and used critical incidence events to emphasize maternal mortality to convince the Governor to support the free MCH services in that state.

Several respondents buttressed how evidence can either enable or constrain advocacy. If the person advocating has compelling evidence such as ugly incidences of what happens during child delivery or health service utilization, this can make advocacy effective. For example, one of the respondents noted: *“if you are going to advocate, it means you advocate on a piece of very firm information and evidence, so if you are advocating on faulty evidence, even if someone listens to you, it may not sound very convincing to attract investment or political will to it” (Developing partner).* Another respondent noted: “*Of course, there is no way that you can do any policy without evidence. For us, you must have evidence to back up our claims and in fact, sometimes we do peer learning of what has worked in other countries” (CSO National).* Such evidence used included what advocates have produced themselves using their data and also as “knowledge brokers” sharing relevant academic data with the decision-makers as one respondent noted, “*If you want the government to put in one naira, you have to tell them what that one naira will achieve based on the data you have” (NGO, National).*

On the other hand, you can have negative effects when there is no concrete evidence or when evidence is biased or skewed. A key constraint is that people engage in advocacy when they are not adequately informed. As noted by a respondent: *It’s a big challenge just like what is happening now, the civil society groups advocating for the implementation of Basic Health Care Provision Fund (BHCPF), so a good number of them do not understand the dynamics of the scheme, so the advocacy is misaligned” (Policy Maker, National).*

#### Networking with powerful and interested champions

Another key contextual influence, which determines the effectiveness of advocacy measures for MCH is engaging key people and elite authorities. Strategic engagements with stakeholders like the minister of health, minister of national planning and minister of finance, legislators, chairman Senate committee on health and chairman house of representative on health and the wife of the Governor after the suspension of funding to SURE-P MCH facilitated the process of sustained concern on MCH both at the national and sub-national level. The different manifestations of their power and influence included the control of resources (the Ministers) policy influence (Governor’s wife, the legislators) and this helps explain specific mechanisms, which these contextual factors triggered. In the words of one of the respondents, *“It was because the first lady (Governor’s wife) was there, and that was a very big driving force and based on that it has succeeded, and we also once in a while have meetings where we invite the wives of the governors … … It was the first lady that we used on this occasion and that was also part of the reasons why the project was moving” (Development partner).*

**Fig. 1:**
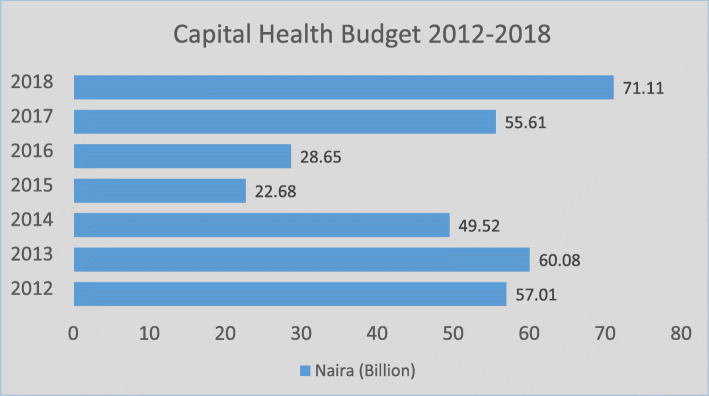
Nigeria Capital Health Budget 2012-2018

Also, the strategic engagements with these stakeholders like the minister of health, minister of national planning and minister of finance, legislators, chairman Senate committee on health, and chairman house of representative on health may have resulted in the increased budget to health. For example, (Fig. 1) shows that there has been an increasing budget for health since 2016. The Capital items within the approved budget range from the provision of vaccines, rehabilitation of hospitals and primary health centers, to the purchase of medical equipment, family planning and reproductive health commodities, interventions in the control of HIV and other diseases, nutrition-related interventions as well as counterpart funding to leverage specific international donor programmes within Nigeria’s health sector [35].

#### Alliance building in MCH advocacy

Group interest and willingness to undertake advocacy on the matter is an important contextual influence on advocacy and a major driver of advocacy activities. Alliance building emerged through a sense of common will, need, and a goal that affects everything that the CSOs and other advocates do. These are examples of mechanisms that a group/collective brings. According to a respondent, *“the fact is the passion, coalitions are formed based on passion. So, the first is the passion that drives the coalition, the second is the ability and the capacity of the coalition and then the unity of purpose. They must have a common vision to be able to achieve any result as a coalition” (CSO National).*

### Determinants of effective advocacy processes

Effective advocacy processes involve alliance brokering (to gain more influence), building relations with media (for adequate dissemination of advocacy agenda and result), champions/influencers (to maximize result), effective mobilization of citizens (for demand creation) and using relevant evidence.

#### Alliance brokering to increase influence

Forming groups is one of the important advocacy processes that can be effective as evidenced by the comment from one of the leaders of a national organization in Nigeria, *“we operate like a big NGO we work with UNICEF, USAID, PATHFINDER to mention but a few in the areas of maternal health” (Professional Group).* It was noted during the interviews that when groups come together, they tend to create a common objective and have a composite position in advocating the government or partners. But when parallel advocacy is done sometimes, it creates much distraction. It was stated that the coalition works better through collaboration, instead of one organization going for it: *“advocacy is better when groups of people come together and have a common vision and through coordinated activity, meet the right people and are given the audience, then they are more likely to achieve their aims. Another thing is having the right person amongst their midst to influence the policy-makers” (Media National).* Although when messages are repeatedly emphasized from different angles and organisations, this can also be an effective tool to consolidate agenda-setting by a sense of social consensus.

#### The media: supporting and engaging in advocacy

A good relationship with the media, which ensured a wider reach and possibly translation of complex messages was an important enabler for the advocacy process by holding public events and disseminating research evidence. Most of the respondents acknowledged that it is difficult to do advocacy without talking with the media as one of our respondents explained: *“we have a relationship with the press and media which is very good, you can’t do advocacy without talking with the media … .. well, most of the activities carried out especially when they concern international health week and all that, the media is usually carried along, immunization days, maternal and child health week, the media is usually involved,” (Policy Maker, State).* This was also echoed by a media expert: *“They call the media when they’ve set the time for the advocacy visits … …… ..So, it wasn’t just “we have an event, come and cover” … … they insisted that the media stay all through with them and I think that’s one time, the media, without knowing it, actually helped in building the message”. (Media National).* Therefore, the media is valued as a key actor in creating an atmosphere of social consensus [36] and concerns that are crucial in supplementing advocacy efforts.

However, this can at times be a double-edged sword since a negative relationship with the media can adversely affect advocacy. One respondent notes that *“one of the challenges we also face in this advocacy is that the media sometimes does not even help when you are not in good standing with them … …*. *the media do not represent those issues the way they are and they don’t give it the appropriate terms” (CSO National).* Misrepresentation and simplification of media messages can constrain advocacy efforts. For example, one respondent noted that a *lot of media people did not understand the Basic Health Care Provision Fund and felt it was the magic bullet to the provision of comprehensive health care and therefore reflected it like that to the populace (Policy Maker State).* Another respondent noted that some media practitioners also misinterpreted the 15% budget allocation health considering it to be *too small given the percentage (Policy Maker, National).* It took the intervention of the policymakers to rectify this misconception.

The media itself also directly engage in advocacy work. In one instance, for example, a symposium on the role of the media in advocating for increased health sector budget for MCH in Nigeria was organized by one of the media organizations, the Health Writer’s Association of Nigeria (HEWAN) and a respondent noted the outcome of this activity was that the media promised more commitment to reporting MCH issues. Also, the 10th quarterly CS-Media forum (overcoming the effect of the recession on maternal health) was held by another media organization, the Development communications network, which brought together health writers, reporters, and civil society organizations to address the effect of the recession on maternal health in Nigeria. A respondent noted that the outcome of the event was that *“the participants agreed to use their various medium to sensitize the need for pregnant women to patronize only registered maternity centers and hospitals headed by qualified personnel, also to adhere to medical advice given on nutrition to prevent complications before and after pregnancy” (Media National).*

In another instance, a media conference on Maternal, Newborn, and Child Health was organized by the Africa Media Development Foundation with participants drawn from the media, government, development partners, NGOs, and CSOs. The conference was aimed at drawing the attention of media practitioners to understand their roles in reducing maternal and child death rates especially in Nigeria. These efforts increased the awareness of key stakeholders to MCH issues.

Several respondents noted specific examples of effective advocacy:

“*There are some advocacy activities we directed at MCH issues. One was about, the Basic Health Care Provision Fund into the budget and having it released as well. (Media State)*

*“There is another advocacy that is on asking for improved funding for health generally to meet up the 15% Abuja declaration” (Media National),*

*“We have seen cases where some line items have been removed from the budget or the funding being cut, but because of our advocacy, those funding were returned and received their appropriate attention”. (CSO State)*

#### Use of Champions, Influencers, and Spouses: Leadership and Elite Gendered Power Dynamics in MCH

The use of champions and influencers in the advocacy process was considered by our participants as an enabler. Once an advocacy issue is identified, those that have the capacity, ability, and passion to drive those issues and their strengths are identified and are used to reach out to the MCH policy-makers and implementers. For example, according to a respondent, “*there was a need to increase the minimum service package for mothers and children in the new Basic Health Care Provision Fund and, the wife of the President was approached and she led the advocacy that resulted in that increased package” (CSO National).* An influencer could be somebody who can influence the decision of another person. A policy champion is usually a powerful individual at the national level (and or state and community levels) and having good connections with different actors and stakeholders including donors and development partners [37]. The policy champion is capable of disseminating, advocating and mobilizing support, and resources. Furthermore, the person can actively facilitate placing problems onto the policy agenda. In the words of one respondent *“you need to have like champions that can mount pressures on government as it is usually difficult for civil servants to say certain things to the government … so you need people like the traditional leaders of the town, the chairmen of ward development committees at the local level” (Policy Maker, State)*. Respected members of society may vary, for instance, between Northern territories in Nigeria where “traditional leaders are members of the elite and so command the respect of political office holders” while in other areas such as Lagos and Benue, community committees are more likely to have influencers members [38].

In MCH, spouses of elite politicians seem to have an important role in brokering policy impact. For example, at the sub-national level (Anambra state), advocacy specifically helped in the entrenchment of MCH on the political and financial agenda. A case in point is related to the activities of the wife of the State Governor. With the backing of the state and local governments, she toured all the primary health centres in the state noting the deficiencies and advocating for safe delivery practices for pregnant women. She further requested the State Governor to provide more funds for MCH. The outcome according to one of the respondents was “*the distribution of maternal delivery kits (MAMA KIT) to pregnant women present and the request to the executive governor of the state to provide more funds for MCH services which he did” (Policy Maker, State).* Also, the mapping showed that she (Governor’s wife) facilitated the passage of the State Primary Health Care Development Agency bill and the release of counterpart funds for the Basic Health Care provision Fund in Anambra state in 2018 for the provision of basic minimum health package.

In another instance, an advocacy meeting on reproductive health was held by the office of the wife of the President of Nigeria to explore how to reduce the high rate of maternal and child mortalities, and child malnutrition in the country. The participants included staff of the Federal Ministry of Health, UNICEF, wives of the 36 state Governors, and the NPHCDA. According to a respondent, the outcome of the meeting was that the Minister for Health pledged Government’s support to the wife of the President’s programme on reproductive health and the Governors’ wives committed to partner with the President’s wife in implementing programmes to reduce maternal and child mortality in their respective states. Another respondent noted that: *“Yes, we had cause to use champions at the community level to mobilize citizens, state-level … … we used role models that can bring attention to all these issues … some were governors’ wives, parliamentary aspirants” (NGO National).* In a society where males have for long dominated public power, the emerging gendered aspect of policy is illustrated in MCH by the explicit role of female spouses. In this policy area, a power shift seems to occur with elite women being recognized and targeted as respected change agents.

## Discussion

This study provided evidence on the mechanism of advocacy activities for sustained prioritization of MCH activities in Nigeria. To understand fully the role of advocacy, three theories were applied. These theories can help to understand the beliefs and assumptions about the way the policy-making process works and identify causal connections to explain how and why a change may or may not occur as a result of advocacy efforts. Combining these theories sheds new light on the effectiveness of advocacy in prioritization of health programmes. They also allow for the transferability of findings from this and how they can be applied in other contexts.

In this study, advocates operated within two of these theories simultaneously and both explained the phenomena being observed. The power politics theory played out in the advocacy for attracting financial commitment, political involvement, policy enactment, and implementation for MCH programs in Nigeria. Our findings showed that advocacy activities were focused on those who had the powers and influence related to MCH. Most of the advocacy groups were seen as capable of influencing decision-makers to take action. These findings corroborate existing literature as shown from a study that assessed the effect of advocacy on implementing a policy of free MCH services policy in Nigeria and showed that this theory was also evident [39]. However, it is pertinent to note that it is not always a success story influencing policymakers because when parallel advocacy is done sometimes, it creates much distraction.

The media influence communication theory also played out in the role and contribution of the media in ensuring sustained political interest in MCH affairs. Media advocacy is aimed at disseminating information through the communications/ media triggering action, such as a change of policy, or altering the views of the public on an issue [40]. A good relationship with the media was an important enabler for the advocacy process in our study as the media and communications activities coupled with advocacy toward decision and policymakers created the support base to take action on the MCH issues. When messages are repeatedly emphasized from different angles and organisations, this can also be an effective tool to consolidate agenda setting. However, a negative relationship with the media can adversely affect advocacy.

Nigeria’s media scene is noted to be one of the liveliest in Africa as radio and Television operate all over the country. For example, all the 36 states of Nigeria run at least one radio network and a television station and most people seem to acquire political information from news media easily [41]. As of 2016, about 86 million Nigerians were online and mobile phones are often used to access the web [41].

Our findings are also supported by the work of Partnership for Maternal, New-born, and Child Health (PMNCH), a global health association. In its 2016–2018 advocacy and communication strategy report, it stated that advocacy and communications are important for designing policy and financial attention to women’s, children’s and adolescents’ health; making sure that latest evidence is made available to all stakeholders, and motivating them to play their role in improving health outcomes. Over the past 10 years, advocacy around maternal, child, and adolescents’ health have resulted in great successes, particularly at the global level [42].

Several authors have demonstrated that among the factors that determine whether an issue is brought to the notice of policymakers or not, is the presence of credible evidence to highlight the severity of the problem to the policy-makers, for example, child mortality rate and maternal mortality ratio [22, 23, 43]. Again these indicators are communicated by the media and used in advocacy activities. This evidences also have the powerful effect of making a hidden issue to be brought to the public and provoking political elites and policymakers to take decisions on the issue.

It is interesting to note here that as opposed to child mortality quantitative data and charts, critical incidence evidence was chosen as adequate evidence for advocacy. Actors’ preferences for different types of evidence for policy have been noted to be influenced by among other things the characteristics of evidence itself, actors’ roles in the evidence process, and their perception of the importance of the evidence [44, 45]. Where there is no such evidence, policymakers and political elites may ignore the issue either because they are unaware of the existence of the problem [37] or such evidence vacuum can be filled by less credible evidence.

In Nigeria for example, the absence of credible evidence contributed to the inertia in the safe motherhood programme. Thus, although reliable data existed to confirm high maternal mortality at that time [46], the evidence and data were not disaggregated into the different States and local governments. As a result, most of the state governors and local government chairmen were unaware of problems in their various areas and avoided putting in place interventions that will reduce maternal mortality [22, 47]. Globally, the development of organized networks of diverse actors expedited the importance given to MCH in the past 10 years. Also, the judicious use of economic and epidemiological evidence by these collaborations of actors influenced attention to policy and encouraged network bonding and globalization processes [48, 49]. However, it is important to note that it is not all the time that evidence works for advocacy, for example, a piece of compelling evidence such as ugly incidences of what happens during child delivery or health service utilization, can make advocacy effective. On the other hand, negative effects occur when there is no concrete evidence or when evidence is biased or skewed. It becomes more obvious therefore that facts or evidence may not work as expected to support advocacy. Advocacy should therefore not devolve completely into emotional persuasion as it might be more of an issue of how the facts and evidence are presented.

In our study, the roles of powerful policy champions and influencers were prominent in the effectiveness of the advocacy process. One of our key study findings is a gendered power shift in MCH with elite women leading on and also advocating for women’s health rights. For example, the wife of the President and Governors’ wives played significant roles in entrenching MCH on the political agenda and strengthening the provision of MCH services, a finding which is similar to earlier studies in Nigeria and other contexts. Some authors established that women’s traditional roles as mothers can be more successful in convincing policymakers because this talks to established normative cultural beliefs about women [50]. Social movements are more likely to achieve change policy outcomes if tailored to local political discourse, gender ideologies, and cultural contexts; and we propose that MCH policy in Nigeria is a “gendered opportunity structure” for policy influence [51]. In pre-colonial Nigeria, particularly in Yoruba and Igbo ethnic groups, women had some economic and political influence strongly linked to their maternal responsibilities, which was further weakened by the colonial period [52]. Male-dominated political administrations were implemented by the British colonialism, negating any indigenous female political power and promoting Victorian values of womanhood that prevented them from participation. Nigerian women, however, were key in resisting and liberating from colonial rule, and nowadays, they have managed to carve out their way into participation in mainstream political movements [53] but they still seem to do so around their socially acceptable roles in society [52].

In a Nigerian study, it was noted that several factors accounted for the success of their advocacy process including the political commitment by the President of the country, the presence of a political elite who provided evidence-based information on maternal and child mortality to policymakers, and involvement of the media and other stakeholders [39]. Furthermore, a Nigerian NGO used the same advocacy mechanism in engaging stakeholders to take part in the family planning advocacy agenda, to increase the use of modern family planning methods [54]. In Tanzania using available evidence health advocates interested in maternal health lobbied for the improved working condition of midwives, by mobilizing affected communities and starting advocacy campaigns all over the country. This drew the attention of members of Parliament and traditional and social media to the poor working conditions of midwives [55].

Finally, the study found out that advocacy was not achieved in silos but through networks of interactions including the community, NGOs, CSOs, international agencies, etc. with influences reaching from national to sub-national and the local level in complex ways as noted elsewhere [56]. Therefore, the coalition works better through collaboration, instead of one organization going for it. For example, such interactions between advocacy organizations for women’s and children’s health rights and government institutions are no longer uncommon in Nigeria. These coalitions have been effective in achieving important policy outcomes, however, their ability to sustain change is determined by whether they can remain as independent pressure groups due to funding and financial constraints and weak democratic processes in Nigeria [57]. Advocating effectively for better Maternal Health and HIV policies, programmes, good leadership, and adequate financing of key public health issues, CSOs can, bring together skills and resources, to project the policy issue [58]. The authors concluded by noting that it is important to put coalition to work by sharing evidence and resources, organizing goals and materials, influencing decision-makers using varied methods, and advocating for improved reproductive health and HIV policies and programmes.

It is important to note that in this study, we did not treat context as a separate set of results but as part of the Context-Mechanism-Outcome (C-M-O) configuration of the articulated programme theory. Therefore, relevant contextual triggers of the mechanisms through which advocacy worked at the State and Federal levels (such as the presence of clear leadership and purpose, joined-up efforts and clear purpose of advocacy effort) were reported as part of the testing of the programme theory.

### Limitations

There are three limitations to this study. First, the participants were mainly stakeholders in maternal and child health who were limited in number. Therefore, it is difficult to generalize the findings. Second, we explored only one State of the Federation to understand the effect of advocacy activities at the sub-national level. However, we believe that our robust methodology enabled us to generate findings that are empirically reliable on how advocacy works, and they reflect what happened in other States during the period of inquiry. We did not assess the effectiveness of advocacy efforts as it was outside the scope of this paper and represents an area for future research. Last, while we reported multiple outcomes of advocacy efforts, our focus remained on primarily advancing the understanding of how advocacy works. This meant that we did not systematically examined the outcomes of all advocacy events, an approach which also reflects the nature of advocacy and often intangible nature of shorter- and longer-term outcomes. However, systematic examination of advocacy effects can be usefully addressed in future research.

## Conclusions

Advocacy comprises a varied range of activities that can be used to make an impact on the policy. Although this can be complex and difficult in many cases, it requires consistency and tenacity for results to be achieved. Realist Evaluation methods are useful in understanding the enabling and constraining factors for the effectiveness of advocacy efforts as well as the mechanisms of how advocacy works. In the context of poor health outcomes, interest from policymakers and politicians in MCH, combined with advocacy from key policy actors and stakeholders armed with evidence, can lead to prioritization and sustained implementation of MCH services. It, therefore, becomes imperative that advocacy activities should be widely supported and encouraged at the national and subnational levels for effective policy enactment and implementation.

Also, in a decentralised health system like Nigeria, where sub-national level actors are not actively involved in the policy process (agenda setting, policy formation) and hence poorly committed to policy implementation, if CSOs and other policy advocates identify and engage key policy influencers through an information campaign and consensus building, this will lead to political and financial commitment at this level which will facilitate and improve MCH policy implementation and health outcomes. Effective advocacy needs to be context-specific and should involve leveraging existing links/relations and using available evidence at the right time for its maximum effect. For effective advocacy, several contextual factors and effective processes need to be considered.

These results help to enrich the existing theories and can be used to advance the advocacy theories by providing deeper insights from the realist perspective into how advocacy works. It is equally important to note that recognizing that different theories exist, and being able to identify when they are overarching theories about how policy change occurs (e.g., Power Politics) or theories about certain tactics (e.g Media Influence communication theory), can help advocates, policymakers, and funders have a common understanding about the differences and similarities in advocacy approaches and policy efforts.

## Supplementary information

**Additional file 1.**

## Data Availability

The datasets generated and/or analysed during the current study are available in the Electronic data stored on the University of Leeds SAN (Storage Area Network) repository, http://library.leeds.ac.uk/info/422/policies/189/university_of_leeds_research_data_management_policy/1. The transcripts of the interviews have been anonymized and are available on this site.

## Abbreviations

ANC: Antenatal Clinic
BHCPF: Basic Health care Provision Fund
CSO: Civil Society Organization
DEVCOM: Development communications network
IDI: In-depth interviews
LMIC: Low and Middle Income Country
MCH: Maternal and child health
MSS: Midwives Service Scheme
MOF: Ministry of Finance
MOH: Ministry of Health
NGO: Non governmental organization
NIENAP: Nigeria Every New Born Action Plan
PMNCH: Partnership for maternal, neonatal and child health
PHC: Primary Health Care
PT: Programme theory
RAMESES: Realist And Meta-narrative Evidence Syntheses Evolving Standards
RMNCAH+N: Reproductive, Maternal, Newborn Child, Adolescent Health + Nutrition
SOML: Saving One Million Lives
SURE-P: Subsidy Reinvestment and Empowerment-Programme
UNICEF: United Nations Children’s Emergency Fund
