# Supplementary material for: What makes advocacy work? Stakeholders’ voices and insights from prioritisation of maternal and child health programme in Nigeria
Source: BMC Health Serv Res. 2020 Sep 18;20:884. doi: 10.1186/s12913-020-05734-0 (PMC7501647; doi:10.1186/s12913-020-05734-0)
Supplement: Supplementary file 1 — Additional file 1. [file 12913_2020_5734_MOESM1_ESM.docx]

**IDI GUIDE FOR ADVOCACY/LOBBYING THEORY TESTING**

| **S/N** | **QUESTIONS** |
| --- | --- |
| **APT1**. **Given the sudden withdrawal of a national MNCH Program and a chronic threat of high MNCH morbidity and mortality, (C) if concerned individuals and groups, form Coalitions and advocate to the Government through Policy Influencers (Champions) (M), this will trigger Government commitment and lead to enactment and funding of sustainable MNCH policies (O).** | |
| **General questions** | |
| 1 | 1. How do you perceive MNCH as a problem in this country (state)?   b. What do you think are the specific problems associated with MNCH in Nigeria (Anambra state for state level interview)?  **Probe for:**   - Existence of indicators, key publications, documents etc. to portray this. - Problems associated with policy formulation, use of evidence, policy implementation |
| **Broader questions to establish/confirm the common starting point about sub-theory** | |
| 2 | **Given the sudden withdrawal of the SURE-P, and given that there is still a need to improve MNCH in the country (C);**  What are the current government commitments/ what is the degree of government commitment to sustain MNCH as a national priority? e.g. New MNCH policies (**Please ask for examples)**   - What policies and programmes have been set up by the government following the withdrawal of SURE-P?   **Note**: **Context**   - What led to the development of the policy/programme?   **Mechanism**   - How was the policy developed? - Who were the key actors involved? - Was evidence used; if so what type of evidence?   **(Expected ) Outcomes**   - Some of the expectations that the policy/programme will achieve   **(Use above to specifically probe theSave One Million Lives [SOML] programme)** |
| 3 | 1. Our discussions with researchers and other stakeholders, suggest that advocating to and lobbying Govt. to give priority to a programme (e.g. SURE-P MNCH) can increase political and economic commitment to that programme.   **What is your opinion of this?**  **b)** From literature we know that Advocacy changes can happen through coordinated activities among a range of individuals with the same core policy belief (Advocacy Coalition) or through a window of opportunity when Advocates can successfully connect components of the policy process (policy window); amongst others.   1. **How does this align with your understanding and opinion of Advocacy?**      1. **Which advocacy and lobbying efforts for Govt. to prioritise MCH are you aware of?**   **Select 1-2 of any events mentioned and explore in depth:, probe for the following:**   - Please describe the process of the Advocacy activity - Which group (s)/individuals conducted the advocacy? - Policy champion (s) - Coalitions among groups/individuals (Names of different coalitions/groups) - Who drove the policy process? - How were you involved in the advocacy process, and what specific role(s) did you play? - How long did the advocacy last? - Any facilitators or constraints to the activities? (contextual factors etc.) - **How can constraints/barriers be mitigated?** |
| 4 | 1. What changes have you noticed in the area of MNCH as a result of these advocacy activities? **(At National and state levels)** 2. **If any changes identified by respondent,** probe for reasons (e.g. timing; media attention; celebrity champions; funds etc.) for attributing these changes to the advocacy/lobbying activities.   **Probe for:**   - Government increase in budget and funding, Human and material resources - Timeliness of government support (timely release of budget etc.) - Enactment of new national and state level MNCH policies/programmes |
| **APT 2-**In decentralized health systems, where sub-national level actors are not actively involved in the policy process(Agenda Setting, Policy formulation)**( C)** and hence poorly committed to policy implementation, if civil society organizations (CSOs) identify and engage key policy influencers through information campaign and consensus building **(M),** this will lead to political and financial commitment at this level which will facilitate MNCH policy implementation and hence, improve health outcomes **(O).** | |
| 5 | 1. In your opinion, who are the key MNCH actors at the sub-national level (Anambra state)?   b. How involved are sub-national actors in the national level MNCH policy process activities prior to implementation (agenda setting, policy formulation)?  c. Where sub-national actors have not been actively involved in the MNCH policy processes, what have been the consequences; in your experience?  **Probe for actors in:**   - State Ministry of Health (SMoH), - State primary Health Care Development Agency (SPHCDA), - Local Government Authorities (LGA) etc.   **Probe for:**   - Specific activities carried out during SURE-P and After SURE-P e.g. National level meetings and workshops prior to SURE-P, representation in technical working groups (TWGs) - Level of involvement in the ongoing MNCH programme-SOML |
| 6 | 1. Which CSOs in the country have actively advocated for MNCH activities, at the national and state levels?   **Probe for specific characteristics:**   - Composition - Key focus of each CSO/group e.g. Policy formulation; implementation; budgetary allocation, etc.   What are the differences between CSO engagement at the a) national and b) state levels? (e.g. differences in facilitators and barriers contextual; economicetc.)   1. What were the processes/ methods of their engagement during MNCH advocacy?   **Probe for:**   - Coalitions with other groups; - Presence/use of policy influencers (champions); - Information campaign - Consensus building - Level of media involvement |
| 7 | 1. What state level **changes** have you noticed in the area of MNCH as a result of these advocacy activities?   **Probe for:**   - Improved state level political and financial commitment to MNCH policy implementation. - Payment of state and local government counterpart funds in subsequent MNCH programmes (probe specifically for SOML) - Timeliness of government support (timely release of budget etc., provision of human and material resources) at state level - Improved relationship between state and national level actors  1. **If any changes identified by respondent,** probe for reasons (e.g. timing; media attention; celebrity champions; funds etc.) for attributing these changes to the advocacy/lobbying activities. |
